# Using whole immune system characterization (immune profiling) to identify immune biomarkers to determine patient selection, dosing, and efficacy of new immune therapies

**DOI:** 10.1186/2051-1426-3-S2-P90

**Published:** 2015-11-04

**Authors:** Michael Gustafson, Yi Lin, Dennis Gastineau, Ian Parney, Allan Dietz

**Affiliations:** 1 Mayo Clinic, Rochester, MN, USA

## Background

Considerable diversity exists in the immune competency of cancer patients even within patients of the same diagnosis. The relationship between the nature and degree of immune suppression and the responses to different targets of immunotherapy will be the key to the rapid implementation and optimal use of these therapies.

## Methods

We have developed a systems based approach to understand immunosuppression in cancer patients by combining comprehensive whole blood quantitative flow cytometry and classical bioinformatics analyses. Our first generation immune profile analysis identified a unique biomarker consisting of the ratio of CD4+ T cells to CD14^+^HLA-DR^lo/neg^ monocytes that correlate with survival rates in over 90 patients with glioblastoma, non-Hodgkin lymphoma, renal cell carcinoma or ovarian cancer^1^. Our second generation technology counts over 150 distinct immune phenotypes including over 25 myeloid phenotypes both stimulating and suppressive and many with no known function^2^. There are 10 PD-1+ and CTLA-4+ T cell combinations, dozens of other T cell phenotypes, as well as cell types rarely included in cancer immunology including granulocyte phenotypes.

## Results

As the analysis is technology and disease agnostic, this approach is being used across our immune therapy program. To date, more than 100 individuals have been typed. As an example we present preliminary longitudinal data from newly diagnosed glioblastoma patients enrolled in a Phase I clinical trial (n=8) receiving a dendritic cell vaccine with concurrent standard of care demonstrating immunophenotypic changes during the course of therapy.

## Conclusions

The data collected from these studies will provide valuable insight in the immunological deficits of patients prior to treatment and phenotypes that contribute to positive or negative responses. This methodology will likely improve various aspects of dendritic cell and other cancer immunotherapies through the optimization of the targeting of specific deficits, timing of delivery, and the development of combinatorial approaches.

ClinicalTrials.gov Identifier: NCT01957956
